# Trauma patients with SARS-CoV-2 in German ICUs during the 2nd wave of the COVID-19 pandemic

**DOI:** 10.1007/s00068-021-01829-3

**Published:** 2021-11-15

**Authors:** Uwe Hamsen, Christian Waydhas, Jörg Bayer, Sebastian Wutzler, Klemens Horst, Frank Hildebrand, Trauma Section of the German Interdisciplinary Association of Critical Care, Emergency Medicine (DIVI)

**Affiliations:** 1 Department of General and Trauma Surgery, BG University Hospital Bergmannsheil, Ruhr University Bochum, Buerkle-de-la-Camp-Platz 1, 44789 Bochum, Germany; 2 Department of Orthopedics and Trauma Surgery, Medical Center—Albert-Ludwigs-University of Freiburg, Faculty of Medicine, Albert-Ludwigs-University of Freiburg, Freiburg, Germany; 3 Department of Trauma, Hand and Orthopedic Surgery, Helios Dr. Horst Schmidt Kliniken Wiesbaden, Wiesbaden, Germany; 4 Department of Trauma and Reconstructive Surgery, University Hospital RWTH Aachen, Aachen, Germany; 5 Medical Faculty, University Duisburg-Essen, Essen, Germany

**Keywords:** COVID-19, SARS-CoV-2, Trauma, ICU, Intensive care unit

## Abstract

**Purpose:**

In January and February 2021, about 4000 severe acute respiratory syndrome coronavirus type 2 (SARS-CoV-2) positive patients were treated daily in German intensive care units (ICUs). The number of SARS-CoV-2-positive ICU patients with trauma, however, is not known and neither whether the trauma itself or COVID-19 causes the critical illness.

**Methods:**

A total of 173 German ICUs, representing 3068 ICU beds, participated in a survey developed by the Trauma Section of the German Interdisciplinary Association of Intensive Care Medicine (DIVI).

**Results:**

Participating ICUs reported an overall 1-day prevalence of 20 and an overall 7-day prevalence of 35 SARS-CoV-2-positive trauma patients in the ICU. Critical illness was triggered by trauma alone in 50% of cases and by the combination of trauma and COVID-19 in 49% of cases; 70% of patients were older than 65 years and suffered from a single injury, predominantly proximal femur fractures. The distribution of patients was comparable regarding the level of care of the trauma centre (local, regional, and supra-regional).

**Conclusion:**

The proportion of trauma patients of all SARS-CoV-2-positive critically ill patients is small (~ 1%) but relevant. There is no concentration of these patients at Level 1 trauma centres. However, the traumatic insult is the most relevant cause for ICU treatment in most of these patients. Regarding a new wave of the pandemic, adequate trauma dedicated resources and perioperative structures and expertise have to be provided for COVID-19 trauma patients.

**Supplementary Information:**

The online version contains supplementary material available at 10.1007/s00068-021-01829-3.

## Introduction

The novel coronavirus (severe acute respiratory syndrome coronavirus-2 (SARS-CoV-2)) pandemic is a burden for global and German health care. The intensive care unit (ICU) resources are of particular concern. The German Interdisciplinary Association of Intensive Care Medicine (DIVI), together with governance partners, implemented an ICU registry. The data of this registry have already been implemented in some studies [1–3]. Daily updates of this registry include cases of coronavirus disease 2019 (COVID-19) patients. The available and occupied ICU capacities are reported from about 1300 hospitals in Germany. Thereby, the registry allows us to identify regional and/or temporal occupancy of ICU beds and, therefore, provides important real-time information for the allocation of resources.

Based on the data of the ICU registry, especially during the first wave of the pandemic in Germany (Spring 2020), a concentration of SARS-CoV-2-positive patients in specialized hospitals was discussed. An associated lack of medical structure and expertise for life-threatening conditions other than COVID-19 was assumed as a potential disadvantage of this strategy. During the second wave of the pandemic in Germany (December 2020 until February 2021), the daily proportion of SARS-CoV-2-positive patients was up to 5700 of 24,000 ICU beds resulting in a serious burden for all hospitals.

However, the ICU registry cannot discriminate between critically ill COVID-19 patients and SARS-CoV-2-positive patients in whom critical illness is caused by other conditions. To evaluate the number of trauma patients who tested positive for SARS-CoV-2 during the second wave, we, therefore, performed a survey including all German ICUs. Characteristics of patients, including an estimate to whether the trauma itself or COVID-19 caused critical illness in these patients, were investigated.

## Methods

### Development of the questionnaire

The Trauma Section of DIVI represents intensivists with specialization predominantly in anaesthesiology and trauma surgery. Experienced experts from the trauma section developed a questionnaire including 12 questions using a Delphi process (Questionnairesupplement), with regard to the clarity and overall representativeness of clinical practice [4]. For example, participants of the survey were requested to estimate the number of SARS-CoV-2-positive trauma cases in the last 12 months. More detailed data on age, diagnoses and whether the need for intensive care treatment were due predominantly to the SARS-CoV-2 infection, trauma alone or the combination of both were requested for patients treated during the day before (1-day prevalence) or the last 7 days before (7-day prevalence) participation in this survey.

#### Sampling method

All medical directors of registered ICUs in the DIVI database were electronically invited to answer the survey using an anonymized web-based platform (http://www.surveymonkey.com). The first invitation was sent on February 2, 2021 and a reminder was sent on February 15, 2021. The closing date was February 19, 2021.

### Frequency statistics

Data on overall ICU-bed capacities and SARS-CoV-2 cases on German ICUs were taken from the DIVI registry [1].

### Statistics

For data analysis, the calculation of descriptive statistics was performed using Microsoft EXCEL Version 16.44 (2019). These statistics summarize details about the sample and provide information about the population from which the sample was drawn. Frequency statistics followed and include absolute frequencies (raw counts) for each category of the discrete variable, relative frequencies (proportions or percentages of the total number of observations), and cumulative frequencies for successive categories of ordinal variables.

## Results

### Descriptive characteristics of survey participants

A total of 173 questionnaires were completed and analysed. This corresponded to a response rate of 9.9%. The 173 participating ICUs represented a total of 3604 ICU beds (high and low care). As the ICU registry of DIVI includes an overall number of 24,000 ICU beds, our survey therewith represented 15% of all ICU capacities. Participants were affiliated with a local (level 3) trauma centre in 34% of cases, a regional (level 2) trauma centre in 34% of cases and a supra-regional (level 1) trauma centre in 20% of cases. In 12% of cases, the hospital was not certified as a trauma centre.

### Frequency statistics

#### Occupancy of ICU capacities over the observation period

During the observation period of the survey between 19,947 (83%) and 20,924 (87%) ICU beds of the aforementioned total number of 24,000 were occupied. There were 4252 SARS-CoV-2-positive patients registered at the beginning of the observation period (February 2, 2021) and the number of cases decreased to 3068 towards the end (February 19, 2021).

### Characteristics of SARS-CoV-2-positive trauma patients

In the last 12 months before the survey, an estimate of 346 SARS-CoV-2-positive trauma patients were admitted to the participating ICUs; 22% of these patients were treated in a level 1 trauma centre, 38% in a level 2 trauma centre and 36% in a level 3 trauma centre.

The overall 1-day prevalence was 20 SARS-CoV-2-positive trauma patients. A minimum of 0 patients and a maximum of 3 patients was reported by a single institution.

**Fig. 1:**
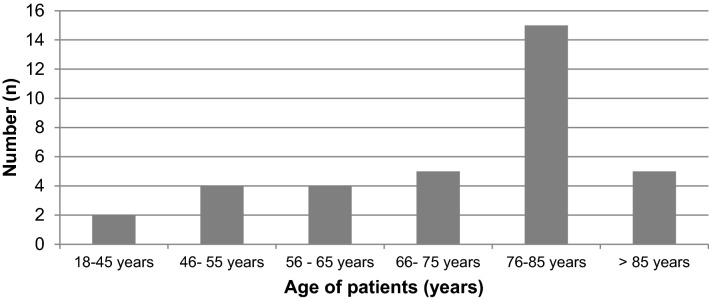
Age pattern of SARS-CoV-2-postive trauma patients in the ICU within 7 days (7-day prevalence)

An overall 7-day prevalence of 35 SARS-CoV-2-positive trauma patients was reported. The majority of these patients was between 76 and 85 years old (Fig. 1); 71% (*n* = 25) of patients suffered an isolated injury, with proximal femur fractures being the most common (*n* = 16). Isolated thoracic trauma was reported in three cases. Six patients were polytraumatized (Table 1). Twenty-two patients were discharged to normal wards, 4 patients died and 9 patients remained in the ICU at the time of the survey. In 50% of cases, the critical illness requiring ICU treatment was caused by the injury and its sequelae alone. In 49% of cases, the combination of trauma and COVID-19 led to critical illness, and only in 1% of trauma patients, ICU treatment was required due to COVID-19 itself.

**Table 1:** Diagnoses of SARS-CoV-2-positive trauma patients, 7-day prevalence

Isolated injuries	*n*
Prox. femur fracture	16
Thoracic trauma	3
Pelvic fracture	2
Traumatic brain injury	1
Spine fracture	1
Fracture of upper extremity	1
Multiple trauma	6
n/a	5

## Discussion

The SARS-CoV-2 pandemic has led to increasing pressure on hospitals and especially on ICU resources. The ICU registry of DIVI has been primarily established to provide real-time information for the allocation of ICU resources. However, the reason for ICU admission and treatment is not documented. Therefore, it remains unknown if the ICU stay is primarily caused by the SARS-CoV-2 infection or by a specific disease or insult (e.g., trauma).

Encompassing 15% of all ICU capacities, we investigated these issues for trauma patients in a representative sample of ICU resources in Germany. Assuming that the participants of our survey represent 15% of all SARS-CoV-2-positive trauma patients, this would mean that about 1–2% of all SARS-CoV-2-positive ICU patients were trauma patients. Without specially focusing on ICU patients, a review including international studies before October 2020 also tried to elucidate the relevance of COVID-19 infections in the trauma population [5]. Although the included studies were too heterogeneous to perform a meta-analysis, the authors were able to conclude that the amount of SARS-CoV-2 trauma patients appears to be small but nevertheless relevant, which is in line with the results of our study.

Interestingly, in only 1% of cases, the critical illness was solely caused by COVID-19, while in all other cases, trauma alone or the combination of trauma and COVID-19 led to critical illness. About half of all patients (49%) were in need of ICU treatment due to the combination of trauma and COVID-19. It is, therefore, likely that COVID-19 worsens the prognosis as compared to trauma alone. In this context, a recent international multicentre study, investigating the relation between SARS-CoV-2 detection and outcome after operation, supported this assumption [6]. Out of 140,231 preoperatively tested patients, 3127 (2.2%) were SARS-CoV-2 positive. There were 17,412 (12.4%) trauma patients included in this study of which 1.1% were SARS-CoV-2 positive and had operation within 2 weeks after testing. Proportion of operations early and very early after testing was markedly higher in trauma patients compared to those with cancer. A surgical procedure within the first weeks after detection of SARS-CoV-2 was associated with a higher mortality (1–2 weeks: odds ratio (OR) 4.1, 3–4 weeks: OR 3.9, 5–6 weeks: OR 3.6). Obviously, this association has to be considered in elective surgery and reconstructive surgery after trauma. However, as the incidence proportions clearly shifted towards emergency operations during the pandemic, the relevance of this finding is limited due to the potentially life- or extremity-threatening character of the injury in the acute phase after trauma [7]. The aforementioned results of our study are important for future discussions about the structure of ICU capacities provided for COVID-19 patients. While major efforts are made to concentrate SARS-CoV-2-positive patients in specialized ICUs or even specialized hospitals, it has to be kept in mind that there is a small but relevant proportion of patients in need of specialized traumatological and perioperative expertise at hand.

Regarding the level of care of the trauma centre, we did not find a concentration of SARS-CoV-2-positive trauma patients in hospitals with a higher level of trauma care. This finding may be explained by the injury pattern of the patients, with a small proportion of young and severely multiple injured patients and a large proportion of geriatric patients over 75 years of age with isolated fractures. In this context, it is remarkable that all hospitals report a maximum of three patients in the 1-day prevalence data, indicating that, for at least the observation period of this survey, no specialized “COVID-19 trauma centre” for a specific region had been established. This suggests that the pandemic-associated burden is a matter of concern for all trauma centres, independent of their level of care. Focusing on this aspect, other diverse studies have investigated the general effects of the pandemic on German trauma centres without reporting on the proportion of critically ill patients or the proportion of SARS-CoV-2-positive trauma patients. In a single-centre study by Popp et al. [8], the authors investigated the number of trauma patients during the first wave (March to May 2020) of the pandemic compared to the same period of the previous years in a single university hospital. They reported a decrease of the number of major trauma patients by 28%, with an associated decrease in workplace accidents, traffic accidents and sports injuries. Comparable developments were also confirmed by international studies [9–12]. However, different studies reported on some specificities. In a study by Wähnert et al. [13], the changes of trauma patient numbers were most pronounced in smaller trauma centres, whereas level I trauma centres even observed a slight increase of emergency cases. Furthermore, for some causes of accidents (assaults, self-inflicted injuries, and injuries associated with home improvement projects), no significant changes or even an increased incidence was described [14]. The authors of the aforementioned studies uniformly concluded that, although the number of trauma patients decreased in the exceptional state of the pandemic, there is still a significant need for functioning structures for the care of trauma patients, as their numbers still remains high. This aspect is of special importance, as Haffer et al. found a substantial reduction of resources in the trauma departments due to the pandemic [15]. They assumed a reduction of about 50% regarding personal and operating room capacities.

### Limitations

There a several limitations to our findings. The response rate to our survey is only 9.9% which might be explained by the fact, that the clinical work load due to the pandemic was so high that the medical staff was too busy to answer this survey. We cannot distinguish between trauma patients without COVID-19-associated symptoms on admission that developed a symptomatic disease during the hospital stay and those patients that suffered an injury while already suffering from COVID-19. This information might be interesting when allocating SARS-CoV-2-positive patients to hospitals or ICUs. The assessment of the cause of critical illness (COVID-19 vs. trauma) was done in a highly subjective manner and we cannot be sure that this estimation would equal an assessment after completion of ICU therapy. Furthermore, using the findings of our survey for prognosis of further waves of the pandemic in Germany, the age distribution of patients might differ a lot as the vaccination of the population in Germany proceeds, starting with the very old and old people.

### Conclusion

Based on this survey, we estimate that 1–2% of all critically ill SARS-CoV-2-positive patients in the ICU are trauma patients. Although most of them are geriatric patients with single fractures, critical illness is mainly caused by the trauma itself or the combination of trauma and COVID-19. There is no concentration of these patients in level 1 trauma or other specialized centres. For the allocation of SARS-Cov-2-positive patients to hospitals and ICUs, it is important to continuously provide substantial structure, resources and expertise for traumatology and perioperative medicine.

## Supplementary Information

Below is the link to the electronic supplementary material.

Supplementary file1 (DOCX 36 KB)

## Data Availability

Data are available from the corresponding author and will be provided to researchers who meet the criteria for access to confidential data.
