# Supplementary material for: Trauma patients with SARS-CoV-2 in German ICUs during the 2nd wave of the COVID-19 pandemic
Source: Eur J Trauma Emerg Surg. 2021 Nov 15;48(2):827–31. doi: 10.1007/s00068-021-01829-3 (PMC8592079; doi:10.1007/s00068-021-01829-3)
Supplement: Supplementary file 1 — Supplementary file1 (DOCX 36 KB) [file 68_2021_1829_MOESM1_ESM.docx]

Supplement: The original questionnaire of the survey in German and translated

| german | translated |
| --- | --- |
| Meine Intensivstation versorgt die Traumapatienten eines   1. Lokalen Traumazentrums 2. Regionalen Traumazentrums 3. Überregionalen Traumazentrums 4. Sonstiges: | My ICU is related to a   1. Level 1 trauma centre 2. Level 2 trauma centre 3. Level 3 trauma centre 4. Other: |
| **Alle Fragen beziehen sich ausschließlich auf die intensivmedizinische Behandlung traumatologischer Patienten** | Questions only include intensive care treatment of trauma patients |
| 1. Haben Sie in den vergangenen 12 Monaten bereits Traumapatienten (s.o.) behandelt, die SARS-CoV2-positiv waren? | Did you treat SARS-CoV-2-positve trauma patients in the last 12 months? |
| 1. Wie viele solcher Patienten haben Sie auf Ihrer Intensivstation behandelt? (ggfs. schätzen)   exakt:  geschätzt: | How many of those patients did you treat?  exactly:  estimated: |
| 1. Wie viele SARS-CoV2-pos. Traumapatienten werden heute auf Ihrer Intensivstation behandelt? | How many SARS-CoV-2 positive trauma patients are treated on your ICU today? |
| 1. Wie viele SARS-CoV2-pos. Traumapatienten wurden in den letzten 7 Tagen auf Ihrer Intensivstation behandelt? | How many SARS-CoV-2 positive trauma patients were treated on your ICU during the last 7 days? |
| 1. Wie alt sind diese Patienten der letzten 7 Tage: 2. 18-30 Jahre: 3. 31-45 Jahre: 4. 46-55 Jahre 5. 56-65 Jahre 6. 66-75 Jahre 7. 76-85 Jahre 8. >85 Jahre 9. Freitext: | How old were those patients of the last seven days?  a) 18-30 years old:  b) 31-45 years old:  c) 46-55 years old:  d) 56-65 years old:  e) 66-75 years old:  f) 76-85 years old:  g) >85 years old:  h) Comments/other: |
| 1. Welche traumatologische Diagnose hatten diese Patienten? | What were the trauma diagnoses of these patients? |
| 1. Bitte schätzen Sie den bei diesen Patienten der letzten 7 Tage den Anteil an den u.g. intensivmedizinischen Behandlungsindikationen. Die Indikation zur intensivmedizinischen Behandlung ergab sich aus 2. Überwiegend aus dem Trauma: n= 3. Überwiegend aus der Operation: n= 4. der Kombination aus COVID-19-Erkrankung und dem Trauma/der OP: n= 5. überwiegend der COVID-19-Erkrankung: n= 6. Freitext: | Regarding those patients oft he last 7 days, please estimate the indication for intensive care treatment. Intnesive care treatment was due to   1. mainly the trauma: n= 2. mainly the operation: n= 3. the combination of trauma/operation and COVID-19: n= 4. mainly COVID-19: n= |
| 1. Wieviele SARS-Cov2-pos. Traumapatienten sind in den letzten 7 Tagen von Ihrer Intensivstation auf eine Normalstation verlegt worden? | How many SARS-CoV-2 positive trauma patients were discharged to normal ward during the last 7 days? |
| 1. Wieviele SARS-CoV2 -pos. Traumapatienten sind in den letzten 7 Tagen auf Ihrer Intensivstation verstorben? | How many SARS-CoV-2 positive trauma patients died on ICU during the last 7 days? |
| 1. Die verstorbenen SARS-CoV2-pos. Patienten sind aus meiner Einschätzung überwiegend verstorben als Folge 2. Des Traumas: n= 3. Der Kombination aus Trauma und COVID-19-Erkrankung: n= 4. Der COVID-19-Erkrankung: n= 5. Freitext: | From my estimation, those non-survivors mainly died due to   1. the trauma: n= 2. the combination of trauma and COVID-19: n= 3. COVID-19: n= 4. others/comments: |
| 1. Haben Sie Anmerkungen/Kommentare? | Do you have any comments? |
